# The impact of high fat diets on physiological changes in euthyroid and thyroid altered rats

**DOI:** 10.1186/1476-511X-12-100

**Published:** 2013-07-12

**Authors:** Venus Welch-White, Norma Dawkins, Thomas Graham, Ralphenia Pace

**Affiliations:** 1 Department of Food and Nutritional Sciences, Tuskegee University, 204 Campbell Hall, Tuskegee, AL 36088, US; 2 Department. of Pathobiology, Tuskegee University, 118 Williams Bowie Hall, Tuskegee, AL 36088, US

**Keywords:** High fat diets, Proplythiouracil (PTU), Liver, Transminases, Altered thyroid

## Abstract

The association of adverse health with high fat intake has long been recognized. However, the lack of research focusing on the interrelationship of thyroid and liver function, and the pathogenesis of a high fat diet leaves these topics poorly understood. The objective of this study was to evaluate and compare the physiological changes in euthyroid and thyroid altered animal model fed saturated and unsaturated high fat diets. To achieve this objective adult male Sprague Dawley rats (n = 100) were fed one of five diets; a control or one of four test diets containing 25% saturated or unsaturated, and 37% saturated or unsaturated fats for a period of eight weeks. Each experimental group consisted of ten euthyroid and ten thyroid altered animals. An altered thyroid state was chemically induced with the addition of 0.05% propylthiouracil (PTU) in the drinking water. Euthyroid animals fed high fat diets increased in body weights and body lengths, compared to thyroid altered animals (P < 0.05). Alanine aminotransferase (ALT) and asparte aminotransferase (AST) levels increased across all experimental groups. HbA1C values and urinary glucose values were within normal range for all animals. Liver morphology showed increased hepatic stellate (ito) and vacuole cells in thyroid altered animals. These findings suggest that altered thyroid status negatively impacts growth and weight gain, and simultaneously affected lipid metabolism, resulting in abnormal liver morphology.

## Background

Dietary fat plays a major role in human nutrition and serves many essential functions. It is necessary to facilitate absorption of fat-soluble vitamins (A, D, E and K) and carotenoids; provides insulation that prevents heat loss, and protects vital organs from shock during normal activities [1]. Diets with adequate energy (30% originating from fat) are sufficient to promote normal growth and normal sexual maturation; conversely, diets that exceed this amount may result in excessive weight gain [2]. Dietary fat intake and excessive caloric intake has been proposed as a causative factor in the development of metabolic syndrome. In this regard, the quantity as well as the quality (primarily) of dietary fat consumed strongly predicts the prevalence and possibility of insulin resistance, type 2 diabetes and atherosclerosis [3,4]. In addition, a high fat diet lowers glucose uptake and inadequately suppresses hepatic glucose production stimulated by insulin [5,6]. High-fat diets have been reported to impair glucose metabolism in rat skeletal muscle, which is the major site of insulin-stimulated glucose metabolism [7]. In the U.S. food prepared away from home is higher in total energy, total fat, saturated fat, cholesterol and sodium, and contains less fiber and calcium and is overall of poorer nutritional quality than food prepared at home. Also, the fat content of at-home food has fallen considerably from 41% of total energy in 1977 to 31.5%, but there has been no change in the fat content of food prepared away from home which remains approximately 37.6% [8]. Higher than necessary energy intake has also been shown to cause whole body and skeletal muscle insulin resistance, hyperinsulinemia, hyperglycemia and, if perpetuated over an extended period of time, could lead to the development of diabetes [7]. Research shows that the fatty acid composition in TGs mainly affects the development of obesity, diabetes and hyperlipidemia [9]. Excessive saturated fat (SFA) consumption promotes lipid storage and inflammation, while polyunsaturated fatty acids (PUFAs) play a protective role by controlling the synthesis and oxidation of SFA. Furthermore, monounsaturated fatty acids (MUFAs) lower hepatic fat content, and improve blood lipid profiles associated with risk of cardiovascular disease [10]. Additionally, dietary fat has also been associated with endocrine and metabolic changes [5,6]; the liver serves as a site for triglyceride and cholesterol metabolism, while the thyroid plays a role in hepatic lipid homeostasis.

Moreover, diseases of thyroid, namely hypothyroidism and hyperthyroidism, constitutes the most common endocrine abnormality in recent years, diagnosed either in subclinical or clinical form. According to a six-year NHANES III Study, the prevalence of hypothyroidism and hyperthyroidism in the population aged 12 and older were 4.6% (0.3% clinical and 4.3% subclinical) and 1.3% (0.5% clinical and 0.7% subclinical), respectively [11]. Risk factors associated with hypothyroidism are clustered within metabolic syndrome and is also characterized by insulin resistance [12]. The influence of high dietary fat intake and the implications of a compromised endocrine system may contribute to the risk of obesity, metabolic syndrome and insulin resistance. Additionally, there is evidence that hypothyroidism may directly affect the liver structure or function. Hypothyroidism has been associated in a few case reports with cholestatic jaundice attributed to reduced bilirubin and bile excretion [13]. In experimental hypothyroidism, the activity of bilirubin UDP glucuronyltransferase is decreased, resulting in a reduction in bilirubin excretion [14]. Thyroid hormones (THs) also oppose the action of insulin and stimulate hepatic gluconeogenesis and glycogenolysis; they up-regulate the expression of genes such as glucose transporter type-4 (GLUT--4) and phosphoglycerate kinase, involved in glucose transport and glycolysis, respectively, thus acting synergistically with insulin facilitating glucose disposal and utilization in peripheral tissues [15]. The liver is the second largest organ in the body and plays a central role in the maintenance of systemic lipid homeostasis. It serves multiple functions including metabolism of lipids and carbohydrates, hormone production, synthesis of clotting factors, and detoxification [16,17]. The morphology of liver, as well as many organs, is directly related to its function [18,19]. Substantial disruption in its anatomy or function may result in the severe alteration of its metabolic roles, which may adversely affect physiological functions. The most common abnormality in liver function tests is a two-to threefold elevation in the alanine aminotransferase (ALT) and aspartate aminotransferase (AST) levels (transaminases). They are associated with inflammation and/or injury to liver cells, a condition known as hepatocellular liver injury. Damage to the liver typically results in a leak of AST and ALT into the bloodstream [20]. The AST/ALT ratio is usually < 1, which can help distinguish non alcoholic fatty liver disease (NAFLD) from alcoholic-related liver disease. When it is > 1 in NAFLD, it suggests an advanced fibrotic stage of disease. In a recent large survey of 12,808 men, ALT-dominant liver disorders were related to obesity, lack of physical exercise, and hyperlipidemia, whereas AST-dominant liver disorders were related to alcohol consumption and diabetes mellitus [21]. A study which evaluated high fat/high cholesterol diets resulted in increased liver weight, fat deposition, inflammation, and fibrosis with increased plasma activity of liver enzymes [22]. Unexplained aminotransferase elevation has been strongly associated with adiposity and other features of the metabolic syndrome [23]. Therefore, the objective of this study was to evaluate and compare the physiological changes in euthyroid and thyroid altered animal model fed saturated and unsaturated high fat diets.

## Results and discussion

### Body weights, Body lengths, Feed intake

The average baseline body weight (d = 0) of animals in all experimental groups ranged from 118 g to 125 g, with no statistical differences among groups. The final body weights (d = 58) of all euthyroid animals were higher compared to the thyroid altered animals across all treatment groups (Table 1). Fat percent had no impact on weight gain; there were no differences between 25 and 37% soy diets and 25 and 37% lard diets in euthyroid animals. It was expected that thyroid altered rats would have significantly higher body weights than the euthyroid animals, however the lower body weight seen in thyroid altered animals may be explained by the influence of thyroid hormones. The thyroid gland plays a critical role in the de novo synthesis of fatty acids and the degradation of lipids and simultaneously influences all major metabolic pathways in the body including those associated with obesity and metabolic syndrome [12]. Alternatively, thyroid dysfunction is often associated with low basal metabolic rate that can influence weight gain. Thyroid hormones impact the metabolism of carbohydrates and fats and influence the synthesis, mobilization and degradation of fats, which is often linked with increased lipoprotein and lipase activity [24]. Sprague Dawley rats have shown varied responses in food intake and weight gain when exposed to high-fat diet. Wang et al. [25] conducted a study where male Sprague Dawley rats were fed high fat (40%) soy oil and lard diets, and there were no significant differences in final body weight, weight gain, and energy intake.

**Table 1:** Final body weights (g) of euthyroid and thyroid altered animals (n = 100)

**Diet**	**Euthyroid**		**Thyroid altered**	
	**Adaptation**	**Final**	**Adaptation**	**Final**
**Control**	120.68 ± 7.15	348.41 ± 22.06 ^a^	121.64 ± 5.18	316.39 ± 17.50 ^b^
**Lard 25%**	119.28 ± 4.70	375.42 ± 13.28 ^ac^	119.23 ± 5.78	319.31 ± 17.90 ^b^
**Soy Oil 25%**	124.92 ± 7.15	390.52 ± 29.99 ^ad^	119.57 ± 6.46	308.48 ± 38.29 ^b^
**Lard 37%**	118.85 ± 6.71	388.07 ± 20.27 ^ac^	123.83 ± 8.43	322.48 ± 15.65 ^b^
**Soy Oil 37%**	121.21 ± 7.02	396.43 ± 21.29 ^ad^	123.13 ± 5.72	320.91 ± 24.17 ^b^

*Means ± SD.

^a, b^ Values within row with different superscript differ at P < 0.05.

^c, d^ Values within column with different superscript at P < 0.05.

The body lengths (Table 2) of the thyroid altered animals was significantly lower than the euthyroid animals (p < 0.05). A possible reason for this divergence may be due to decreased thyroid function during the animal’s rapid growth phase, as a result of the early introduction (six weeks of age) of PTU treatment. The rapid growth phase occurs between 8 and 14 weeks of age. This may explain decreased body weight and body length, as the growth period may have been interrupted. Thyroid hormones are critical for normal bone growth and development. Biochemical studies have shown that these hormones can affect the expression of various bone markers in serum, reflecting changes in both bone formation and reabsorption, and increases alkaline phosphatase and osteocalcin in osteoblasts [26]. The epiphyseal growth plate is made of several key characteristics including cartilaginous, bony, and fibrous components, which act together to achieve longitudinal bone growth. The epiphyseal growth plate is the target element for longitudinal growth [27]. Christian and Trenton [28] reported a study in which PTU was administered to Long-Evans rats daily from birth until 30 days of age. They reported impaired growth and reduced body weights. Their results are in agreement with the present study and support the conclusion that the administration of PTU contributed to decreased body weights, organ weights and body lengths.

**Table 2:** Final body lengths (cm) of euthyroid and thyroid altered animals

**Diet**	**Group**	
	**Euthyroid**	**Thyroid altered**
**Control**	43.00 ± 1.52 ^b^	40.72 ± 0.98 ^c^
**Lard 25%**	43.78 ± 0.72 ^ab^	40.29 ± 1.62 ^c^
**Soy oil 25%**	44.26 ± 0.93 ^a^	41.06 ± 1.12 ^c^
**Lard 37%**	44.48 ± 0.80 ^a^	40.70 ± 1.84 ^c^
**Soy oil 37%**	44.64 ± 0.70 ^a^	40.67 ± 0.84 ^c^

*Means ± SD.

^a, b, c^ Values across rows and within columns with difference superscript differ at P < 0.05.

The feed consumption throughout the experiment was lower (Table 3) in all thyroid altered rats when compared to euthyroid rats (P < 0.05). This reduction in feed consumption substantially contributed to the lower weight gain, observed in the thyroid altered rats. Amin et al. [29], suggested that the triiodothyronine (T3) hormone plays a critical role in appetite control. PTU functions to inhibit the mechanism of thyroid iodide peroxidase (TPO), which is needed to produce the T3 hormone. The results in the present study would support the role of T3 contributing to appetite control, as food intake among thyroid altered animals was significantly lowered than euthyroid rats. The T3 values (Table 4) revealed a trend of higher levels of T3 hormone in all thyroid altered animals. There were significant differences (P < 0.05) in the control group and the 25% lard group across the euthyroid and thyroid altered animals from the remainder of the experimental groups. The higher values may be attributed to feedback loop inhibition in which the hormone was not being utilized [26]. Damage to thyroid glands was confirmed through histopathological evaluation (data not shown). Furthermore feed intake was not influenced by the type or amount of fat in the diets. It would appear that thyroid status had a greater impact than dietary composition.

**Table 3:** Average feed consumption (g) of euthyroid and thyroid altered animals n = 100

**Diet**	**Group**	
	**Euthyroid**	**Thyroid altered**
**Control**	1448 ± 21.75 ^a^	1266 ± 72.15 ^b^
**Lard 25%**	1431 ± 4.92 ^a^	1138 ± 55.03 ^c^
**Soy oil 25%**	1428 ± 13.26 ^a^	1190 ± 89.15 ^c^
**Lard 37%**	1419 ± 10.77 ^a^	1126 ± 40.34 ^c^
**Soy oil 37%**	1410 ± 19.20 ^a^	1119 ± 53.15 ^c^

*Means ± SD.

^a, b, c^ Values within rows and columns with difference superscript differ at P < 0.05.

**Table 4:** T3 values in euthyroid and thyroid altered animals

**Diet**	**Group**	
	**Euthyroid**	**Thyroid altered**
**Control**	3.05 ± 1.49 ^cd^	4.6 ± 1.59 ^ab^
**Lard 25%**	5.74 ± 0.93 ^a^	4.02 ± 1.70 ^bc^
**Soy oil 25%**	0.84 ± 0.59 ^e^	1.78 ± 1.01 ^de^
**Lard 37%**	0.95 ± 0.91 ^e^	1.68 ± 1.13 ^de^
**Soy oil 37%**	1.68 ± 1.14 ^de^	2.52 ± 1.32 ^d^

*Means ± SD.

^a, b, c, d, e^ Values across rows and within columns with different superscript differ at P < 0.05.

### Lipid profile

The normal range of total cholesterol in rats is 20 – 92 mg/dL. The lipid profile (Table 5) revealed elevated total cholesterol in the thyroid altered animals compared to the euthyroid animals; however, there were no statistical differences between the treatment groups. These results may be due to the impairment of the thyroid gland necessary to metabolize cholesterol, and confirms the effectiveness of the PTU treatment. High density lipoprotein (HDL) values were higher in the control diet groups. Triglyceride levels were not significantly different between euthyroid and thyroid altered rats, regardless of diet (P < 0.05). Low density lipoprotein (LDL) values were the highest among thyroid altered animals. The liver serves as a major site for cholesterol and triglyceride metabolism, and the thyroid hormones play an integral part in hepatic lipid homeostasis. Thyroid hormones increase the expression of LDL receptors on the hepatocytes and increase the activity of lipid lowering liver enzymes, resulting in a reduction in low density lipoprotein levels [30]. Thyroid hormones also increase the expression of apolipoprotein A1, a major component of high density lipoprotein [13]. In rats, thyroid hormones stimulate hepatic cholesterol biosynthesis, decrease hepatic triglyceride secretion, increase bile formation and excretion of cholesterol, and decrease intestinal cholesterol absorption. In the case of hypothyroidism, an increase in plasma cholesterol concentration may be expected [31]. Estrany et al. [32] reported that the consumption of a high fat diet containing monounsaturated (46.7%) and saturated (43.3%) fat decreased serum triacylglycerols and total cholesterol in male rats when compared to levels in a control diet containing 24.1% monounsaturated and 20.7% saturated fat, respectively. The findings of this study also revealed variations in levels of total cholesterol and triglyceride, although differences were not significant among treatment groups. The HDL values were higher in the experimental groups which consumed diets high in monounsaturated fat (MUFA), yet thyroid altered animals showed a trend of decreased HDL values. Jenkins et al. [33] reported that a higher intake of monounsaturated fat may raise high-density lipoprotein (HDL) cholesterol which is consistent with the findings of the present study.

**Table 5:** Lipid profile of euthyroid and thyroid altered animals (n = 100)

**Experimental group**	**Euthyroid**				**Thyroid altered**			
	**Total cholesterol**	**HDL**	**TG**	**LDL**	**Total cholesterol**	**HDL**	**TG**	**LDL**
**Control**	87.2 ± 20.56 ^a^	53.4 ± 16.89 ^ab^	60.7 ± 15.11 ^a^	45.9 ± 20.57 ^ab^	92.3 ± 13.56 ^a^	57.1 ±18.15 ^ab^	54.6 ± 10.36 ^a^	57.5 ± 33.03 ^a^
**Lard 25%**	90.9 ± 21.54 ^a^	72.1 ± 17.71 ^a^	57.8 ± 10.10 ^a^	40.1 ± 15.54 ^abc^	93.0 ± 24.99 ^a^	56.4 ± 34.69 ^ab^	61.1 ± 11.59 ^a^	48.8 ± 25.85 ^abc^
**Soy oil 25%**	90.2 ± 13.24 ^a^	50.2 ± 9.78 ^b^	50.5 ± 11.64 ^a^	29.9 ± 15.82 ^bc^	88 ± 5.25 ^a^	56.0 ± 13.71 ^ab^	54.9 ± 16.61 ^a^	24.2 ± 6.51 ^c^
**Lard 37%**	84.3 ± 8.67 ^a^	54.3 ± 14.71a ^b^	58 ± 15.25 ^a^	21.8 ± 11.65 ^c^	89.4 ± 7.72 ^a^	47.2 ± 21.81b ^ab^	65.2 ± 30.37 ^a^	35.6 ± 14.53 ^bc^
**Soy oil 37%**	88.6 ± 14.89 ^a^	39.9 ± 7.99 ^b^	57.8 ± 22.08 ^a^	37.2 ± 9.31 ^bc^	89.6 ± 13.21 ^a^	40.6 ± 16.49 ^b^	51.9 ± 19.28 ^a^	38.6 ± 19.23 ^abc^

*Means ± SD.

^a, b^ Total Cholesterol values across rows and within columns with difference superscript differ at P < 0.05.

^a, b^ HDL values across rows and within columns with difference superscript differ at P < 0.05.

^a, b^ TG values across rows and within columns with difference superscript differ at P < 0.05.

^a, b, c^ LDL values across rows and within columns with difference superscript differ at P < 0.05.

### Liver enzymes

Liver enzymes, AST and ALT were elevated across all treatment groups (Table 6). The normal ranges of ALT and AST are 17.5 – 30.2 U/L and 45.7 – 80.8 U/L, respectively. ALT levels were highest among euthyroid rats fed unsaturated fat diets compared to the control rats (p < 0.05). The groups with the highest levels of AST consumed the 25 and 37% soy diets. Subsequently, these rats had the highest body weights. The elevation of these liver enzymes values may be indicative of some liver impairment, or possibly damage. Liver damage resulting from underlying cellular death is often associated with cholestasis, drug-induced injury and obesity [34]. The liver and the thyroid gland are intricately connected in lipid metabolism as well as in the maintenance of homeostasis. The findings of this study revealed increased ALT and AST values; this may suggest that the role of dietary fat and thyroid status influence the levels of these transaminases. Panchal et al. [22] evaluated high fat/ high cholesterol diets, and reported increased liver weight, fat deposition, inflammation, and fibrosis with increased plasma activity of liver enzymes.

**Table 6:** ALT and AST serum values in euthyroid and thyroid altered animals (n = 100)

**Diet**	**ALT**		**AST**	
	**Euthyroid**	**Thyroid alt**	**Euthyroid**	**Thyroid alt**
**Control**	43.3 ± 24.73 ^ab^	38.5 ± 38.50 ^b^	151.6 ± 85.46 ^a^	94.4 ± 47.99 ^a^
**Lard 25%**	46.9 ± 13.20 ^ab^	39.4 ± 5.70 ^ba^	115.1 ± 69.18 ^a^	112.3 ± 47.81 ^a^
**Soy oil 25%**	51.5 ± 11.30 ^ab^	46.9 ± 18.82 ^ba^	148.7 ± 96.39 ^a^	153.9 ± 102.30 ^a^
**Lard 37%**	46.7 ± 9.06 ^a^	48.11 ± 14.89 ^ba^	128.6 ± 65.53 ^a^	149.8 ± 105.54 ^a^
**Soy oil 37%**	53.8 ± 12.72 ^a^	48.4 ± 10.84 ^ba^	143.9 ± 88.59 ^a^	121.7 ± 62.99 ^a^

*Means of Average Reading Values.

^a, b^ ALT Values across rows within columns with difference superscript differ at P < 0.05.

^a^ AST Values across rows and within columns with difference superscript differ at P < 0.05.

### Histopathological evaluation of the liver

**Figure 1:**
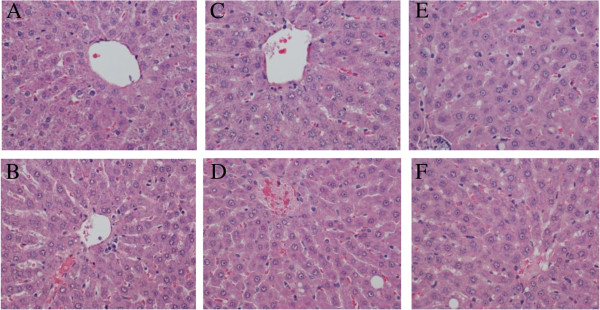
**Histopathological evaluation of liver in Rats fed Lard Based Diets. (A)** Represents a normal liver with normal morphology from a euthyroid animal which consumed a control diet. **(B)** Represents a normal liver with normal morphology from a thyroid altered animal which consumed a control diet. **(C)** Represents a normal liver in a euthyroid animal with normal morphology, which consumed a 25% lard diet. **(D)** Shows liver from a thyroid altered animal which consumed 25% lard diet. There is evidence of +1 ito cells and a hepatocyte with the presence of two small cytoplasmic vacuoles. **(E)** Represents the liver from a euthyroid animal which consumed 37% lard diet that shows the presence of 3 ito cells (+4) and multiple vacuoles (+3). **(F)** Represents the liver from a thyroid altered animal which consumed 37% lard diet that shows the Presence of ito cells (+4) and vacuoles (+2).

**Figure 2:**
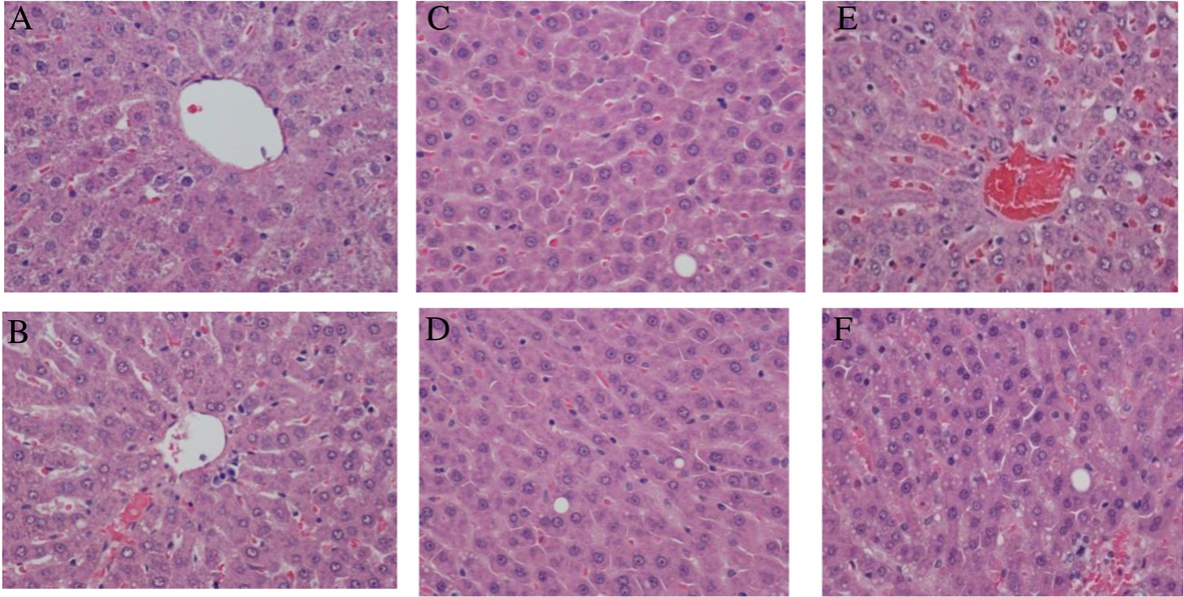
**Histopathological evaluation of liver in Rats fed Soy Oil Diets. (A)** Represents a normal liver with normal morphology from a euthyroid animal which consumed a control diet. **(B)** Represents a normal liver with normal morphology from a thyroid altered animal which consumed a control diet. **(C)** Represents a liver in a euthyroid animal which consumed a 25% soy diet and shows the presence of 2 ito cells, with a grade of (+2). **(D)** Shows liver from a thyroid altered animal which consumed 25% soy diet and displays the presence of 2 ito cells (+2). **(E)** Represents the liver from a euthyroid animal which consumed 37% soy diet and displays the presence of ito cells (+3) and vacuoles (+2). **(F)** Represents the liver from a thyroid altered animal which consumed 37% soy diet that shows the displays the presence of 1 ito cells (+2) and small vacuoles (+4).

The results of the histopathological evaluation of livers from rats are shown in Table 7. The tissues showed an increased presence of hepatic stellate (ito) cells and vacuoles in the thyroid altered animal compared to the euthyroid animals. The thyroid altered animals had an occurrence of vacuoles of 60% or greater across all treatment groups. Morphological changes were observed in the 37% fat dietary groups, as well as in the thyroid altered animals. Hepatic stellate (ito) cells are naturally occurring lipid containing cells and play a key role in the pathogenesis of hepatic fibrosis [35]. Fibrosis is the essential pathophysiologic consequence of chronic liver injury, and it represents the common underlying mechanism for hepatic insufficiency [36] Figure 1 and 2.

**Table 7:** Summary incidence of abnormal histopathological liver changesin euthyroid and thyroid altered animals

**Diet**	**Euthyroid**		**Thyroid altered**	
	**Ito cells (≥2+)**	**Vacuoles**	**Ito cells (≥2+)**	**Vacuoles**
**Control**	0%	20%	20%	80%
**Lard 25%**	0%	30%	50%	70%
**Soy 25%**	20%	30%	60%	80%
**Lard 37%**	10%	0%	50%	80%
**Soy 37%**	0%	20%	30%	60%

It is suggested that hepatic stellate (ito) cells play a key role in hepatic fibrogenesis, regardless of the underlying etiology [36]. A pathological grade of +2 or higher hepatic stellate (ito) cells denotes abnormality and may suggest comprised lipid metabolism. Abnormal fat vacuoles are indicators of increased intracellular lipid, which may account for abnormal synthesis, utilization, and/or export of fat [37]. These vacuoles are not naturally occurring and their presence may infer liver abnormality. Non-alcoholic fatty liver disease (NAFLD) is a liver disease defined by both clinical (non-alcoholic) and histopathological characteristics [21]. NAFLD ranges from steatosis and hepatic insulin resistance to advanced fibrosis and cirrhosis. Currently, NAFLD is considered as the hepatic manifestation of the metabolic syndrome, triggered by mechanisms including inflammation, lipid overload and oxidative stress in adipose tissue and liver [38]. Pagadala et al. [39] reported a higher prevalence of hypothyroidism in patients with NAFLD compared to controls. The histopathological results of this study revealed that increased hepatic stellate (ito) cells and vacuoles may indicate precursors of the development of NAFLD, which is currently under further evaluation.

## Conclusion

The oral administration of PTU successfully induced hypothyroidism. The results revealed that thyroid altered animals had smaller bodies and decreased body weights. The liver and the thyroid gland work intricately together in lipid metabolism as well as maintain homeostasis. Thyroid altered animals had a trend of higher cholesterol compared to the euthyroid animals. ALT and AST values were higher for the thyroid altered groups. This may suggest that the role of dietary fat and thyroid status influence the level of these transaminases. Additionally there were increased occurrences of hepatic stellate (ito) cells and vacuoles in the liver of thyroid altered animals compared to the euthyroid animals, across all treatment groups. Chemically induced hypothyroidism negatively impacted fat metabolism and growth, which contributed to increased lipid accumulation in the liver.

## Methods

### Chemicals

Propylythiouracil or PTU (6-propyl-2-thiouracil) was obtained from Sigma Aldrich (St. Louis, MO). HDLC kits were obtained from Abcam (AbCam Cambridge, MA). Total cholesterol and triglycerides kits were obtained Wako analyzing kits (Wako Chemicals USA, Richmond, VA).

### Animal diets

Diets were obtained from Purina Lab Diets/Test Diets (Richmond, IN). The diets were designed to be isocaloric and isonitrogenous. The saturated fat diets contained lard, while unsaturated fat diets contained soy oil. The USDA dietary guidelines recommend dietary fat of 25 to 30%, of daily energy intake were taken into consideration when designing the diets. The percentages of fat from calories in the diets were 37% (lard and soy), 25% lard and soy), and 12% (modified AIN93 which served as the control). The fat levels in the diets represent high, moderate and low.

### Animals and Experimental design

**Figure 3:**
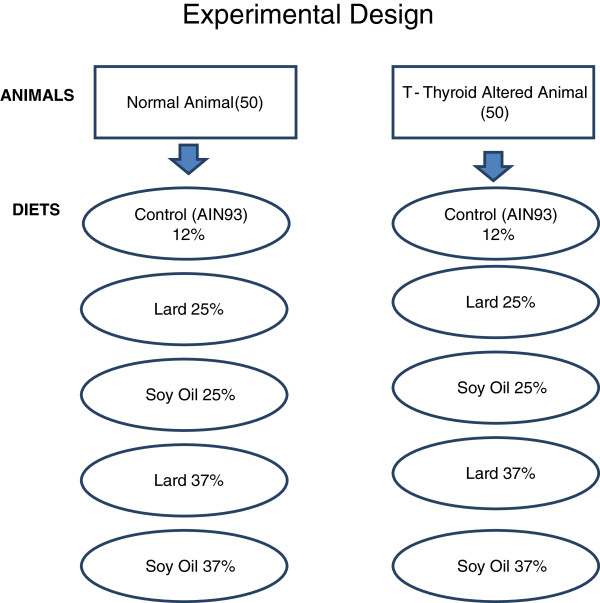
**Experimental groups and test diets.** Five of the experimental groups were comprised of normal or euthyroid rats, and the remaining five experimental groups contained thyroid altered animals. Each diet of the five diets were fed to a normal (euthyroid) group as well as a thyroid altered group. Saturated fat diets contain lard as the fat source, while unsaturated diets contain soy oil.

One hundred, 30 – 35 day old male Sprague Dawley rats, with a mean weight of 100 g were obtained from Harlan Sprague Dawley Laboratories, Inc. (Indianapolis, IN). The animals were housed two per stainless steel cage (60 cm × 60 cm × 15 cm), quarantined for 7 -d (fed rodent lab chow) and acclimatized to the respective diets for 7-d, after arrival at the university’s, College of Veterinary Medicine Comparative Medicine Resource Center. The room temperature was 21 ± 1 C with relative humidity of 50 ± 5% and a light/dark cycle of 12 hours (light 8:00 – 20:00). Ten animals were weighed and randomly assigned to one of ten experimental groups (n = 100); (Figure 3). Euthyroid and thyroid altered animals were pair fed one of the five diets (control , 25% lard, 25% soy, 37% lard and 37% soy) for 8-weeks and had access to water *ad libitum* throughout the duration of the study. PTU was administered orally and continuously to five experimental groups (n = 50) in the drinking water at 0.05% (w/v) for 6 weeks. Corn syrup was added to the drinking water to increase palatability in the PTU- treated groups, and as a control in the non-treated groups. PTU inhibits the mechanism of thyroid iodide peroxidase (TPO) resulting in a chemically-altered thyroid state the models [40]. Feed consumption was measured daily and the animals were weighed weekly. Body lengths were measure at the conclusion of the study. The rats were fasted overnight on the final day of the study, prior to euthanasia from overexposure to CO_2_. All experimental procedures were conducted according to the approved protocols and guidelines of the university’s Animal Care and Use Committee.

### Blood, tissue collection and Histopathology

Blood samples were collected via cardiac puncture, from each animal, for serum (EDTA containing tube), plasma (heparin containing tube) and whole blood analysis (BD Diagnostics, Franklin Lakes, NJ). Liver tissues were harvested for histopathological analyses. The right lobe of the liver was excised and placed in a 10% neutral buffered formaldehyde solution for further analysis. The tissues were sectioned, embedded, mounted and stained with hematoxylin and eosin. The liver tissues were evaluated for the presence of accumulated fat and general morphology. All slides were then evaluated and graded by a veterinary pathologist. The animal carcasses were disposed of by incineration at the Post-Mortem Building at the university’s College of Veterinary Medicine.

### Lipid profiles

The plasma concentrations of HDL-C were measured enzymatically using Abcam analyzing kits, as described by manufacturer (AbCam Cambridge, MA) and total cholesterol and triglycerides were enzymatically measured using Wako analyzing kits (Wako Chemicals USA, Richmond, VA). Low density lipoprotein (LDL) cholesterol was estimated indirectly with the Friedewald equation.

### Statistical methods

Data were analyzed using the GLM procedure (SAS Inst. Inc., Cary, NC) to compare the ten treatment combinations. When the omnibus F-test indicated statistical significance (p < 0.05), the treatment means were separated using Duncan’s New Multiple Range Test. In addition, selected contrasts were used to determine the statistical significance of the difference between groups that received varying types and levels of fats. These contrasts were conducted among dietary fat levels and thyroid status. Data are presented as means ± SD.

## Abbreviations

ALT: Alanine aminotransferase; AST: Asparte aminotransferase; HDL: High-density lipoprotein; LDL: Low density lipoprotein; MUFA: Monounsaturated fat; NAFLD: Non-alcoholic fatty liver disease; PTU: Propylthiouracil; T3: Triiodothyronine; TPO: Thyroid iodide peroxidase.

## Competing interests

The author declares no competing interests.

## Authors’ contributions

VW carried out the study, data collection and analysis and drafted the manuscript. ND, TG, and RP were responsible for study design. All authors read and approved the final manuscript.
